# Which emotion regulation strategy is efficient for whom? Reappraisal and suppression efficiency for adaptive and maladaptive personality profiles

**DOI:** 10.1111/jopy.12948

**Published:** 2024-05-27

**Authors:** Elena Trentini, Elise Dan‐Glauser

**Affiliations:** ^1^ Institute of Psychology University of Lausanne Lausanne Switzerland

**Keywords:** emotion regulation, Five‐Factor Model, Maladaptive Personality Trait Model, personality profiles, psychophysiology, reappraisal, suppression

## Abstract

**Objective:**

This study aimed to explore the efficiency of different emotion regulation strategies, specifically reappraisal and suppression, in relation to adaptive and maladaptive personality profiles.

**Background:**

Personality conditions emotions and influences emotion regulation. Of the available regulation strategies, reappraisal (reinterpreting the situation) is described as an efficient strategy, whereas suppression (not displaying the experienced emotion) carries higher physiological and cognitive costs. Little is known, however, about the influence of personality on these efficiencies.

**Method:**

We tested the personality structure of 102 participants (Mean_age_ = 20.75, *SD*_age_ = 2.15), based on the Five‐Factor Model and the Maladaptive Personality Trait Model. Experience, expressivity, and physiological arousal were recorded during the viewing of emotionally charged positive and negative images while participants reappraised, suppressed, or viewed the images without regulating their emotions.

**Results:**

We identified two clusters for adaptive personality (“Adaptive Resilient” and “Anti‐resilient”) and two for maladaptive personality (“Maladaptive Resilient” and “Under‐controlled”). The major finding was for emotional experience in maladaptive personalities, where reappraisal was efficient in the Maladaptive Resilient profile, while none of the strategies brought relief in the Under‐controlled profile.

**Conclusion:**

This study, which systematically contrasts personality and efficiency of emotion regulation strategies, is one of the first attempts to refine the understanding of how personality influences the emotional regulation process.

## 1 INTRODUCTION

Emotions are a fundamental part of people's daily life as they influence our thoughts and our reactions to situations. Emotions are often regulated in order to adequately react to the environment. Within the multitude of possible emotion regulation strategies, reappraisal and suppression are some of the most studied. Nonetheless, their efficiency in regulating emotions is still debated, and prior studies led to contrasting results. One of the factors that may impact the efficiency of emotion regulation strategies is personality, which seems to be strongly associated with emotions. Personality can be viewed as an individual and unique constellation of thoughts, feelings, and behaviors, causing specific responses to events (Costa et al., 1995; Wiggins & Pincus, 1992). These repetitive patterns in response to a stimulus are defined as personality traits (Avia, 1997). Of the many existing personality taxonomies, the most established and universally recognized is McCrae and Costa (1997) Five‐Factor Model (FFM) for its stability, heritability, consensuality (Goldberg, 1990; McAdams, 1992), and predictive utility (Costa & McCrae, 2008; John & Srivastava, 1999; McAdams & Pals, 2006). Recently, based on the FFM model, another Five‐Factor Model has been created: the Maladaptive Personality Trait Model (MPTM), which describes personality from a more symptomatic point of view (Freilich et al., 2023; Wright et al., 2012). In this study, the aim was to evaluate the efficiency of reappraisal and suppression in association with personality, both from an adaptive and maladaptive perspective. In particular, we wanted to determine which strategy might be more efficient for which personality profile.

### 1.1 Emotion and emotion regulation

While emotions are notably hard to define, we chose to base our study on the definition given by researchers working in the field of appraisal theories of emotions (Frijda, 1988; Moors et al., 2013). According to them, emotions can be defined as short‐term multifaceted phenomena, which stem from people's interpretation of the current situation (Scherer et al., 2001). They consist of changes in several responses, such as experiential, expressive, and physiological ones (Grandjean et al., 2008; Scherer, 2005; Shuman & Scherer, 2015). Experience refers to how a stimulus is consciously lived (Scherer, 2001). Expressivity consists of non‐verbal communication, such as mimicry or gestures, aiming at communicating affective states to others (Gross & John, 1995). Finally, physiological arousal comprehends the emotion‐related body changes, such as respiration pace variation, heart rate changes, and variation in electrodermal activity. These parameters are sensitive to emotional states and responsible for preparing the body for subsequent actions. Studying these three main responses gives the opportunity to analyze, from different perspectives, the comprehensive emotion reaction that a stimulus provokes. Emotions are specifically characterized by frequent occurrences throughout the day and, more importantly, by their rapid onset and short duration. Most of the time, they are modified by emotion regulation (ER) processes, governed by social rules or individual preferences (Izard et al., 2008; Prosen & Vitulić, 2014). ER can be automatic or conscious (Gross, 1998) and includes emotional strategies that individuals can use to manage and influence their emotions (Cole et al., 1994; Ford & Troy, 2019; Gross, 1998; Pollock et al., 2016). There are many reasons to implement ER strategies: from seeking to increase positive emotions and decrease negative ones (hedonic emotion regulation, Kobylińska & Kusev, 2019) to achieving context‐specific goals (Eldesouky & English, 2019; Gross, 2014). In the emotional unfolding, several ER strategies are present, as described in the Process Model of Emotion Regulation (Gross, 1998, 2001, 2014).

Previous studies have defined the efficiency of ER strategies in terms of their adaptability, classifying them into adaptive and maladaptive strategies. On the one hand, adaptive strategies are characterized by goal‐directed regulation, reduction in negative emotions, improved resistance to pain or interpersonal relationships (Aldao & Nolen‐Hoeksema, 2012). On the other hand, maladaptive strategies are linked to negative consequences such as negative or unchanged affective states and an increased risk of psychopathological disorders (Aldao & Nolen‐Hoeksema, 2010). Based on the adaptive‐maladaptive framework, previous studies have yielded contrasting results, indicating that adaptive strategies are not always beneficial and maladaptive strategies are not always negative. A striking example of these many discrepancies is the contrast between reappraisal and suppression.

### 1.2 Efficiency of reappraisal and suppression

Among the strategies described by Gross (1998), reappraisal and suppression are the most studied. Reappraisal can be defined as a cognitive manipulation leading to a reinterpretation of the situation (Purnamaningsih, 2017) before a response is given (Goldin et al., 2008). Suppression occurs later (Gross & Thompson, 2007) and is intended to reduce emotional responses to a stimulus (Gross, 1998).

The primary benefit of *reappraisal* is its general ability to reduce negative experience and expressivity without cognitive or physiological costs (Gross et al., 2006; John & Gross, 2004). Habitual implementation of reappraisal is also associated with higher well‐being (Kobylińska et al., 2020; Kobylińska & Kusev, 2019) and reduced distress (Gross, 1998). John and Gross (2004) indicated that reappraisal needs fewer cognitive resources than other strategies due to its relative early occurrence in the emotional process; while Levenson (2003) suggested that, unlike other strategies, reappraisal does not lead to a rebound effect, triggering an increase in physiological arousal. However, the literature is not unanimous about its beneficial effects. When faced with a strong emotional event, reappraisal is less likely to be attempted due to the difficulty of reinterpreting the situation in the heat of the moment. In such cases, the strategy of situation selection could be more beneficial (Webb et al., 2018). On a physiological level, and except for heart rate, reappraisal has been shown to have little effect in decreasing physiological parameters (Mohammed et al., 2021) and in high‐stress situations, the implementation of reappraisal does not reduce cortisol levels (Zhan et al., 2017). Reappraisal's efficiency can also be influenced by individual differences that may decrease or increase certain emotional responses. As Efinger et al. (2019) reported, people with a high trait‐anxiety level did not show reduced physiological arousal when reappraisal was performed.

*Suppression* is traditionally considered as a maladaptive strategy. It was reported not to provide subjective relief (Gross et al., 2006), and it seems to require more cognitive resources to maintain the emotional reduction (Kobylińska & Kusev, 2019). Additionally, suppression is counterproductive in reducing physiological arousal, with higher physiological costs (Levenson, 2003; Olatunji et al., 2017). Nonetheless, it has been shown to be more efficient in reducing expressivity during negative emotions than reappraisal (Goldin et al., 2008; Levenson, 2003). At the experiential level, Meyer et al. (2012) found also that the habitual application of suppression led neither to adverse consequences on positive affects nor to an increase in negative affects. On a physiological level, suppression was also found to provoke increased parasympathetic activity, with a longer interbeat interval (Lemaire et al., 2014), and it was shown to reduce anxiety and pain with little physiological cost (Braams et al., 2012; Brockman et al., 2017).

Given the contrasting results within strategies, it is possible that other factors may play a role in the regulation process and moderate its efficiency. This could be the case of personality (Timmermans et al., 2009). Individuals presenting different personality trait levels may either implement ER strategies in different ways (Purnamaningsih, 2017; Wang et al., 2009), or present a lack of available resources to implement a given strategy. Studying personality types in association with ER strategies may reveal that certain strategies are more efficient in some individuals than in others, explaining the discrepancies observed so far. We tested this hypothesis by focusing on two main personality models: the Five‐Factor Model and the Maladaptive Personality Trait Model.

### 1.3 Two complementary personality models

In the literature, personalities are often divided between adaptive and maladaptive. Previous literature indicated a partial alignment between the traits of the Five‐Factor Model for adaptive personality and the Maladaptive Personality Trait Model for maladaptive one. However, both models do not totally overlap. The use of both models thus allows a more comprehensive overview of the individuals' personality.

#### 1.3.1 The Five‐Factor Model (FFM)

The FFM is a dominant model of personality, principally because it is reliable across cultures (McCrae & Costa, 1997) and reports robust generality of traits (Goldberg, 1990; McAdams, 1992). The theory includes five main personality traits: (1) *Neuroticism* (N), characterized by negative emotionality, introversion, anxiety, and low emotional stability (Barańczuk, 2019; Bienvenu et al., 2004); (2) *Extraversion* (E), correlated with sociability, assertiveness, and positive emotionality, and defined as the ability to experience and show positive affects (John & Gross, 2007); (3) *Openness to experience* (O), characterized by open‐mindedness to feelings, new ideas, or experiences (McCrae & John, 1992); (4) *Agreeableness* (A), related to interpersonal personality characteristics, and behavioral tendencies to establish positive relationships with others (John & Gross, 2007; Tobin et al., 2000); and finally (5) *Conscientiousness* (C), related to the will to achieve goals and prudence, but also to discipline and dedication (Barańczuk, 2019; McCrae & John, 1992; Reisenzein & Weber, 2009).

#### 1.3.2 The Maladaptive Personality Trait Model (MPTM)

Previous research has highlighted the association between FFM traits and psychological disorders, finding an influence of personality on pathological disorders. For example, compared with other traits, individuals with high N are more likely to suffer from depression and anxiety (Han et al., 2021), and, in combination with other traits at the lower end of the continuum, personality disorders (PD), such as borderline, schizotypal, and dependent disorders (Hopwood et al., 2013). However, research has shown that not all PDs are associated with FFM traits (Krueger et al., 2012; Watson et al., 2013). The MPTM has therefore been proposed as an alternative approach to personality from a pathological perspective, focusing on maladaptive variants of FFM traits, with an emphasis on dimensions associated with psychopathological disorders (Pollock et al., 2016; Watson et al., 2013). The MPTM five traits are: *Negative affectivity* (the tendency to experience negative emotions frequently), *Detachment* (introversion and social isolation), *Antagonism* (the tendency to engage in aggressive and dominant behavior), *Disinhibition* (impulsivity and sensation seeking), and *Psychoticism* (the tendency to have illogical thought patterns, Pollock et al., 2016; Wright et al., 2012). As examples of relationships with PD, antisocial PD may be related to *Antagonism* and *Disinhibition*, while avoidant PD is related to *Negative affectivity* and *Detachment* (Coker et al., 2002; Watson et al., 2013).

Research has supported the alignment between the MPTM and the FFM (Krueger & Markon, 2014; Thomas et al., 2013; Yan et al., 2021), indicating a strong relationship between *Negative affectivity* and N, *Disinhibition* and low C, as well as between *Antagonism* and A (Suzuki et al., 2015; Watson et al., 2013; Widiger et al., 2018). However, traits such as *Psychoticism* and *Detachment* correspond less clearly with the FFM (Watson et al., 2013). Given the lack of complete alignment between the MPTM and the FFM, using both models provides a more comprehensive view of individuals' personalities and may help to better frame the diagnosis and subsequent psychotherapeutic choice (Bach et al., 2016).

### 1.4 Personality and emotion regulation

As mentioned, personality can have an influence on emotions. Indeed, some personality traits are also often referred to as *emotional dispositions* (i.e., the predisposition to experience emotions, Reisenzein et al., 2013). For example, in the FFM, N can be defined as the predisposition to experience negative emotions. E includes positive affects as an essential subcomponent, and O is more related to the ability to experience a broader range of feelings (Reisenzein & Weber, 2009). C is associated with positive emotions elicited by agency in the environment (Shiota et al., 2006) and A is related to positive emotions if they are related to interpersonal relationships (Reisenzein & Weber, 2009; Shiota et al., 2006).

A lot of research has been conducted to date about what strategies were used in daily life and how much these were used, based on the individuals' trait levels. Pollock et al. (2016) suggested that high‐N individuals tend to have fewer resources for ER and experience more negative emotions and relationships. N individuals therefore attempt to reduce the negative experience as quickly as possible (Hughes et al., 2020). Hence, situation selection (Purnamaningsih, 2017) and suppression (Barańczuk, 2019) are frequently used by these individuals to regulate their emotions. On the contrary, extraverted people are more likely to use situation modification and reappraisal (Barańczuk, 2019; John & Gross, 2004; Purnamaningsih, 2017), due to their greater access to ER strategies and their ability to understand and convert emotions into more positive ones (Kokkonen & Pulkkinen, 2001). Individuals high in O tend to be more optimistic about situations and frequently use reappraisal (John & Gross, 2007), situation modification, and distraction (Barańczuk, 2019). Individuals high in C are more likely to regulate their emotions by selecting or modifying the situation, as well as by using distraction to achieve their goals (Barańczuk, 2019; John & Gross, 2007). Finally, individuals with high A scores regulate their emotions based on interpersonal relationships and situations (John & Gross, 2007; Purnamaningsih, 2017). Therefore, they would be more comfortable using reappraisal and making cognitive efforts to control negative emotions such as anger (Kobylińska et al., 2020). Regarding the association between ER and MPTM, all maladaptive traits are generally related to higher emotion dysregulation (Abdi & Pak, 2019). In particular, individuals with high levels of *Negative affectivity* showed low use of ER strategies (Pollock et al., 2016), while highly impulsive individuals have difficulty applying reappraisal (Rogier et al., 2020). In addition, individuals with high levels of *Detachment* tend to limit emotional expression (Abdi & Pak, 2019), suggesting that such individuals might be more comfortable with regulating emotions through suppression. Thus, both adaptive and maladaptive models showed an association with ER strategy use, but less is known about whether these strategies are actually beneficial to individuals.

Regarding reappraisal or suppression efficiency depending on certain traits, research has provided only partial evidence. Regarding the FFM, the successful use of reappraisal in individuals high in O has been demonstrated (Morawetz et al., 2017), as well as in psychotic patients (Opoka, Sundag, et al., 2021). With respect to maladaptive traits, acute delusional PD patients have been shown to successfully regulate their negative emotions using reappraisal and distraction (Opoka, Ludwig, et al., 2021). In contrast, Scheffel et al. (2019) reported a lack of relationship between ER success and personality traits. Despite the growing interest in studying the efficiency of ER through the lens of personality, research in this area is still in its infancy and, to our knowledge, no study has yet tested the influence of personality on ER strategy efficiency in the three main components of emotion response (experience, expressivity, and physiological arousal).

### 1.5 Using a person‐centered approach and working on profiles

Initially, personality psychology was primarily guided by a variable‐centered approach, that is, focusing on each of the single FFM traits (Sârbescu & Boncu, 2018), which allows the convergence of similar associations that can include the entire population. Recently, however, personality research has moved toward a person‐centered approach, that is, the use of personality profiles, based on the idea that individuals would be better represented by the respective level of the traits and their interrelation than by traits alone (Alessandri et al., 2014; De Fruyt et al., 2002; Udayar et al., 2020). This allows to categorize individuals into different subpopulations, where each subpopulation has a specific pattern of traits (Howard & Hoffman, 2018; Udayar et al., 2020).

Regarding adaptive personality, among the different possible combinations of adaptive personality traits, three profiles have emerged as the most stable: Resilient, Under‐controlled, and Over‐controlled (Alessandri et al., 2014; Block & Block, 1980). The Resilient profile is characterized by high adaptive behavior and social skills, with low levels of N and high levels in all other traits. The Over‐controlled profile has high levels of N and C, and low levels of E, and it is related to high emotional instability. Finally, the Under‐controlled profile has high impulsivity, seeks immediate gratification (Sârbescu & Boncu, 2018) and has low levels of C and A but high levels of E and N (De Fruyt et al., 2002; Rammstedt et al., 2004; Sârbescu & Boncu, 2018). Yin et al. (2021) also identified two other profiles: the Anti‐resilient profile and the Ordinary profile. The former is characterized by high levels of N and low levels of all other traits, while the second profile has average levels of all traits.

With respect to the MPTM, some main profiles with this taxonomy started to emerge. Rossi et al. (2021) aimed at exporting the main profiles previously identified within the FFM (Resilient, Under‐controlled, and Over‐controlled) to MPTM profiles in a clinical population. Resilient profile had low levels of all maladaptive traits (but higher in *Negative affectivity*); Over‐controlled profile presented high levels of *Negative affectivity* and low levels of *Antagonism*; and Under‐controlled people showed high levels in all traits. Bastiaens et al. (2021) used a more exploratory approach on a nonclinical population, establishing five different profiles based on the MPTM: Confident‐disagreeable, Anxious‐detached, Anxious‐agreeable, Resilient, Under‐controlled, and Very resilient. According to Bastiaens et al. (2021), among the profiles found, the Resilient profile is defined by overall low levels of maladaptive traits, while the Under‐controlled profile is characterized by overall high levels of all traits, particularly in *Disinhibition*, *Psychoticism* and *Negative affectivity*. These results on Resilient and Under‐controlled profiles are similar to those of Rossi et al. (2021).

### 1.6 The present study

As previously mentioned, emotions can be regulated in different ways and the efficiency of ER strategies can affect several emotional responses. However, the strategies' efficiency is still debated, and contrasting results are still present. We suppose that the origin of these result discrepancies could be caused by other factors such as personality, since it has several common associations with ER. Based on a person‐centered approach, we could further argue that belonging to a personality profile may be associated with different efficiency levels of ER strategies (see also John & Gross, 2007; Purnamaningsih, 2017). Our main question, therefore, was to determine ER's efficiency in relationship with adaptive and maladaptive personalities. We tested the efficiency of reappraisal and suppression strategies toward negative and positive emotions while measuring emotional responses (experience, expressivity, and physiological arousal).

To test efficiency, we used a specific calculation of parameter change, namely the Difference Index (DI), which has already been used by Thuillard and Dan‐Glauser (2021) and Trentini and Dan‐Glauser (2023). By placing the results of the unregulated condition as a baseline, the DI gives a direct measure of the strategy efficiency, positive DI signaling strategy efficiency, a DI‐value of 0 signaling no emotional changes with respect to an unregulated condition, and a negative DI signaling increased emotional arousal when using a regulation strategy (hence a counterproductive effect). We defined the strategy as an efficient one when it helped to significantly decrease all the parameter levels, except for heart rate. The only other exception was for the respiration amplitude (RA) parameter, which was calculated with reversed scores. Indeed, for RA, a decrease is usually linked with negative images (Gomez et al., 2004) and an increase seems to be related to a more positive state (Van Diest et al., 2014). Therefore, for this parameter, an increase was interpreted as efficient and, vice versa, a decrease was interpreted as inefficient, for both negative and positive images.

Given the novelty of the study, we made several exploratory assumptions, especially for the ones related to maladaptive profiles, since this specific field is still in its expanding phase. All of the following hypotheses were formulated by taking into account an interaction between the efficiency of ER strategies and personality profiles as an overarching hypothesis to test.

With respect to the adaptive profiles:

(H1a) For experience, and given the adaptive nature of reappraisal and its correlation with profiles high in O, A, and E traits (Purnamaningsih, 2017), it was expected that this strategy would trigger a stronger decrease in negative emotions and an increase in positive emotions compared with suppression in the same personality group. Contrarily, profiles with high levels of N were expected to have more benefits in decreasing negative emotions with suppression than with reappraisal (Barańczuk, 2019). For positive emotions, in the interaction with personality, reappraisal was expected to increase positive experience more than suppression in profiles with high levels of O, A, and E and low levels of N. Evidence demonstrating that suppression, regardless of emotional intensity, results in fewer positive emotions supports this hypothesis (Li et al., 2020).
(H1b) For expressivity, since profiles with high‐N values are more correlated with suppression (Barańczuk, 2019), and that the latter resulted in a bigger decrease in expressivity (Levenson, 2003), it was expected an interaction and that this strategy would be more efficient in decreasing negative expressivity than reappraisal, always in people high in N. With respect to positive emotions, it was expected that profiles with high E, O, and A would show an increase in positive expressivity during reappraisal (Barańczuk, 2019; Gross & John, 1995) but not during suppression.
(H1c) With respect to physiological arousal, it was expected that, in interaction with personality, reappraisal would not reduce physiological parameter values in profiles where N was high. This hypothesis was driven by past results showing that reappraisal was not successful in reducing physiological arousal in people with high trait‐anxiety (Efinger et al., 2019). Since anxiety is one of the aspects that features N (Bienvenu et al., 2004), we paralleled Efinger et al. (2019) results with N levels. A similar effect was hypothesized for suppression in people high in N since this strategy generally leads to an increase in physiological levels (Levenson, 2003).

With respect to maladaptive profiles:

(H2a) For experience, we expected that reappraisal would better reduce negative experience than suppression in profiles where *Psychoticism* was high, as Opoka, Sundag, et al. (2021) reported. For positive experience, since people with high *Negative affectivity* are less used to employ strategies (Pollock et al., 2016), we expected that reappraisal would not be efficient for them, leading to a decrease in positive emotions.
(H2b) Regarding expressivity, and based on the results from Pollock et al. (2016), we expected that profiles high in *Negative affectivity* would show higher expressivity when the participants are confronted to negative images during suppression than during reappraisal. However, this was not expected for profiles with a high *Detachment* component, which were expected to decrease expressivity toward negative pictures more strongly with suppression than with reappraisal (Abdi & Pak, 2019). For positive expressivity, profiles with low maladaptive traits were expected to express greater positive expressivity during reappraisal than during suppression.
(H2c) For physiological arousal, we expected that reappraisal would decrease all physiological parameters more strongly than suppression in profiles with low maladaptive traits, probably because they are less influenced by the maladaptive aspect of personality.

## 2 METHODS

### 2.1 Participants

We tested 102 first‐year psychology students (78 females and 24 males) ranging in age from 18 to 32 years old (Mean_age_: 20.75, *SD*_age_: 2.15). A‐priori sample determination with power analysis presented the need of *N* = 72, specific for ANOVA, power at 0.80, *η*_*p*_^2^ = 0.045, and *α* = 0.05, according to effect sizes found in similar studies in our laboratory and Faul et al. (2007). However, we augmented the number of participants by 40% to counteract data loss. Fourteen participants were dropped from the study for not showing up at the laboratory. The experiment was conducted in French, so participants had to be native French speakers or have excellent French skills. Left‐handed people (Bourne, 2008), pregnant women (Ghorbani‐Marghmaleki et al., 2019; McDonald et al., 2021), people who use drugs (Kober, 2014), medication or alcohol (Petit et al., 2015), and participants with mood disorders (Joormann & Gotlib, 2010; Ma, 2015) or an anxiety diagnosis (Amstadter, 2008; Cisler et al., 2010) were excluded from the study. Psychology students recruited into the study were rewarded with credit points. For this study, the data pool coincided with Trentini and Dan‐Glauser (2023).

### 2.2 Measures

#### 2.2.1 Personality measures

To assess personality, we used the 60‐item version of the NEO‐FFI (a short form of the NEO‐PI‐R, Costa & McCrae, 1992b, 2008, 2009) and the short form of the PID‐5 (PID‐5‐SF, 100 items, Krueger et al., 2012; Thimm et al., 2016). The NEO‐FFI tests FFM traits, has high reliability and test–retest validity, and is appropriate for both control and clinical populations (Anisi et al., 2012; Costa & McCrae, 2008). The PID‐5‐SF assesses MPTM traits, and shows satisfactory reliability (Maples et al., 2015) as well as internal consistency (Thimm et al., 2016). Both surveys were presented in their French versions, which were found to be valid and equivalent to the original versions (Rolland et al., 1998; Roskam et al., 2015).

#### 2.2.2 Emotion

Considering that ER strategies may have a differential impact on emotional responses (Efinger et al., 2019; Matsumoto et al., 2008; Mauss et al., 2005), the three emotional components were tested individually.

##### Emotion experience

During the study, participants continuously rated their levels of positive or negative emotions. They used a rating dial (BIOPAC Systems, Goleta, CA, USA), moving a slider along a line recording voltage on a scale from 0, very negative, to 9, very positive (Dan‐Glauser & Gross, 2011; Hughes et al., 2020). The use of the rating dial permits a continuous assessment of the subjective experience during the experiment, preserving the affective response (Waugh et al., 2011) while affecting neither the emotional experience nor the attention toward the stimuli (Hutcherson et al., 2005).

##### Emotion expressivity

To test expressivity, the activity of facial muscle regions was recorded using bipolar surface electromyography (EMG). Two muscle regions were recorded: the left *Corrugator supercilii* and the left *Orbicularii Oculi*. Activity of the *Corrugator* region is generally related to negative expressions (e.g., frowning), whereas activity of the *Orbicularii* region is related to positive expressions (Germain & Kangas, 2015; Tamm et al., 2020). Electrodes were standard 4‐mm Ag‐AgCl sensors, placed according to the recommendations by Fridlund and Cacioppo (1986). The skin was cleaned with alcohol and rubbed with Nuprep® gel (Weaver and Cie). Electrodes were filled with Signagel (Parker Laboratories Inc.) and placed on the corresponding location on the face (Dan‐Glauser & Gross, 2015).

##### Physiological arousal

Physiological arousal was particularly important to consider, given the association between personality traits and such responses (Bibbey et al., 2013; Stemmler & Wacker, 2010). For example, N is generally related to higher cardiovascular activity, while individuals with high E tend to show lower cardiovascular reactivity (Jonassaint et al., 2009). Electrocardiography (ECG), skin conductance level (SCL), and respiration were all recorded in the present study for several reasons. Electrocardiography and respiration assessed the overall activity of the autonomic nervous system (as they include the influence of both the sympathetic and parasympathetic nervous systems), whereas SCL is known to be primarily influenced by the sympathetic nervous system. Electrocardiography and SCL are particularly important for detecting physiological changes due to emotional reactions (Dan‐Glauser & Gross, 2015; Van Doren et al., 2021). With respect to SCL and personality, it has been found that high‐N individuals are more reactive in SCL than low‐N individuals (Coles et al., 1971). We chose to assess respiratory activity as it contributed to the cardiovascular system activity (Kreibig, 2010; Lorig, 2007).

Electrocardiography was recorded with three sensors in a variant of the Einthoven configuration. One was placed approximately 5 cm below the lower rib on the left side of the abdomen. A second electrode was placed just below the right clavicle, along the clavicle midline. A third electrode, which served as a ground, was placed at the level of the cervical C7 vertebra. SCL was recorded using two pre‐gelled disposable Ag/AgCl sensors placed on the thenar and hypothenar eminences of the palm of the nondominant hand. Respiration was tested using two noninvasive recording belts, one around the waist (abdominal) and the other on the chest (thoracic, Ritz et al., 2002). All parameters and sensors were recorded and amplified with MP150‐compatible modules from Biopac Systems (Goleta, CA, USA). All acquired channels were sampled at 1000 Hz.

For another study, finger pulse (recorded with a photoplethysmography transducer placed on the index finger of the left hand) and skin temperature (measured with a probe placed on the left little finger) were also recorded but are not analyzed further here.

#### 2.2.3 Control variables and other measures

To get a more complete picture of the participants' characteristics, other parameters were controlled for. Before coming to the laboratory, they had to complete the Edinburgh Handedness Inventory (Oldfield, 1971) to assess their right‐handedness. The inventory tests participants' handedness by asking them which hand they use for certain tasks (such as opening a jar or holding a knife). The score, based on a scale between −100 and +100, averaged 93.55 (*SD*: 10.2) in the present study. After completing the laboratory test, they completed several surveys, which were used in another study but not analyzed further here: the Positive and Negative Affect Schedule (PANAS, 20 items, Bouffard et al., 1997), the Toronto Alexithymia Scale (TAS, 20‐items, Bagby et al., 1994), the Patient Health Questionnaire (PHQ‐4, 4 items), the Berkeley Expressivity Questionnaire (BEQ, 16 items), and the Difficulties in Emotion Regulation Scale (DERS, 36 items).

### 2.3 Stimuli

The use of images to elicit positive and negative emotions has proven to be effective and reliable (Codispoti et al., 2001; Ellard et al., 2012), and we therefore used such a paradigm. We presented 144 images (72 negative and 72 positive). The images were grouped into eight categories (four positive and four negative) with 18 images each. The four positive categories included images of babies, panoramas, sports, and cute animals. The four negative categories included snakes, spiders, suffering animals, and suffering humans. One hundred thirty‐one images were selected from the Geneva Affective Picture Database (GAPED, Dan‐Glauser & Scherer, 2011). In addition, we validated 13 novel sport images that had positive valence and high arousal through an online pilot test. During the experiment, emotional stimuli were presented for 8 s.

### 2.4 Conditions

All participants were asked to perform one of three tasks while viewing emotional images: reappraisal, suppression, or the unregulated condition. For the reappraisal condition, the instructions were: “*Adopt a neutral, detached attitude toward the image. Think of yourself as a scientist considering the scene analytically*.” (Efinger et al., 2019); for the suppression condition, the instructions were: “*Observe the image and report what you feel but do not let the emotion affect your face and physiological reactions*.” (Gross & Levenson, 1993); while for the unregulated condition, the instructions were “*Observe the image and let your emotion come and go naturally. Let yourself feel*.” (Gross & Levenson, 1997).

### 2.5 Procedure

**FIGURE 1:**
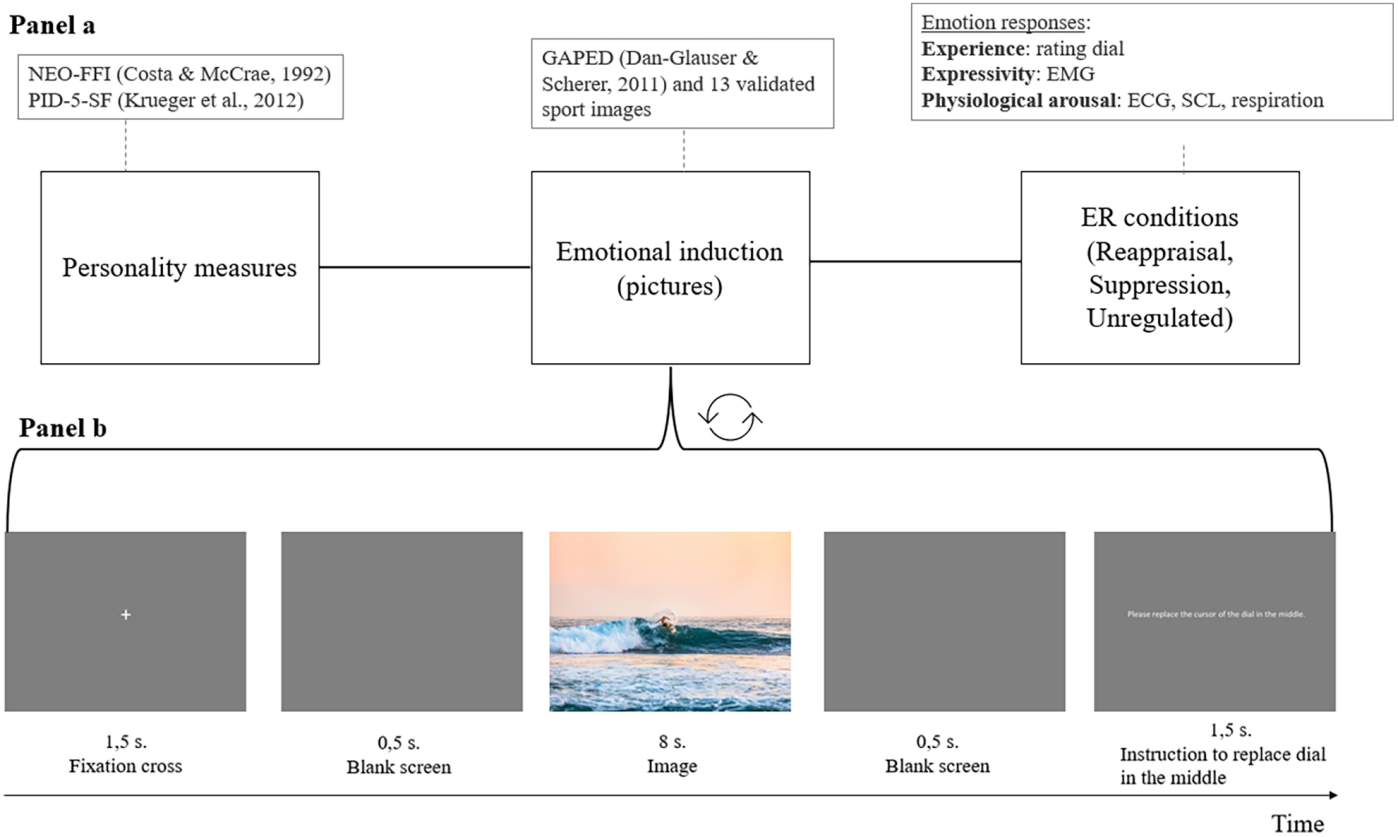
Summary of the main phases of the experiment, with a focus on the trial sequence. Panel a represents the main factors of the study and how they were tested or implemented in the study. Panel b is a focus of a typical trial sequence.

The protocol was approved by the UNIL‐SSP ethics committee (approval number: C_SSP_112020_00006). Part of this protocol having been published elsewhere (Trentini & Dan‐Glauser, 2023), only a summary of the procedure will be presented here (Figure 1, Panel a).

Before coming to the laboratory, participants were asked to complete the Edinburgh Handedness Inventory (Oldfield, 1971). Once this was verified, they were invited to the laboratory. There, they were first informed about the study, its benefits and risks and gave their informed consent. Then, after placing all the physiological sensors, they started the experiment. All instructions and information were given on a computer screen. During the experiment, they had to apply ER strategies, namely reappraisal, suppression, and an unregulated condition. Emotions were elicited by using a picture‐viewing paradigm. We randomly presented 144 pictures, 72 positive. and 72 negatives. The study consisted of nine blocks of 16 pictures, three blocks for each strategy. At the beginning of each block, participants read instructions about the type of strategy they needed to implement. For each trial, participants saw, in sequence, a fixation cross, a blank screen, the image, and then a blank screen again (Figure 1, Panel b). In addition, during each trial, participants were asked to rate their emotional experience using a rating dial placed in front of them. After this task (which lasted approximately 45–50 min), the body sensors were removed, and participants were debriefed by being explained the ER strategies that they applied during the test and, more broadly, our research expectations regarding emotion unfolding. After the laboratory session, participants were asked to complete a final task, which consisted of completing online personality and control questionnaires, accessible through a specific URL given at the end of the laboratory session. This task was completed within 7 days of the laboratory experiment. At the end of the experiment, participants were thanked for their participation with credit points and fully debriefed about the importance of assessing personality within the study of ER efficiency.

### 2.6 Data reduction

All data were processed with Acqknowledge 4.4 (Biopac Systems, Goleta, CA). Recorded channels were filtered to increase the signal‐to‐noise ratio (20–500 Hz for EMG, 0.5–35 Hz for ECG, and 0.05–1 Hz for respiration), and data artifacts were manually scanned and corrected (Efinger et al., 2019; Thuillard & Dan‐Glauser, 2020, 2021). To limit tonic response effects, changes in physiological and expressive channels for each trial were compared with a 3.5‐s baseline extracted from the period before each picture viewing (Thuillard & Dan‐Glauser, 2021). Thus, each trial had this baseline period, which was used to normalize the response and obtain the relative change in parameters after each image presentation. Parameter changes over the entire viewing period of each image (8 s) were analyzed. Trials were grouped by conditions, and each of the regulated conditions was compared with the unregulated condition to analyze strategy efficiency (see Section 2.7).

#### 2.6.1 Emotional experience

Ratings were exported to obtain average values for each trial. The cursor position before each trial (i.e., the position to which the participant returned the cursor after the previous image) was considered the starting point for calculating the emotion intensity for that trial. Any value below this position was considered a negative feeling and any value above this position was considered a positive feeling (“negative” and “positive” labels were written on the dial). Ratings were transformed into an emotion intensity scale extracted as percentages, representing the distance the cursor traveled from its 0 point (starting point) to the position reached on each side during each trial. The data for each of the valences thus ranged from 0 = no emotional experience to 100 = extreme intensity of emotion during the trial.

#### 2.6.2 Emotion expressivity

EMG signals were rectified and smoothed (5 Hz) before being averaged for each trial. Given the large variability in contractile capacity of each individual, each EMG value was then expressed as a percentage of contraction relative to the corresponding trial's antecedent level (voltage recorded for a given time frame/voltage recorded during the 3.5 s preceding the trial × 100, De Wied et al., 2006; Van Boxtel, 2010). Negative expressivity was measured with the *Corrugator* site values, whereas positive expressivity was measured with the *Orbicularii* site values.

#### 2.6.3 Physiological arousal

Heart rate (HR) was calculated from the ECG channel by transforming the interbeat interval (duration between successive R waves). The skin conductance level was exported as mean values for each trial. Nineteen participants had to be removed because they were non‐responders, that is, individuals showing no phasic response in their skin conductance level (Gatti et al., 2018; Kredlow et al., 2017), making their response irrelevant to the trial‐by‐trial examination of physiological reactivity. Respiratory rate (RR) and RA were calculated for each trial. RR was obtained by converting the duration of cycle intervals to a number of cycles per minute (c/min). RA was interpolated using the difference in Volts between the point of maximum inspiration and the point of maximum expiration. Considering the positive correlation between thoracic and abdominal signals in RR and RA, *r* = 0.32–0.56, *p* < 0.001, the values from these sites were averaged for RR and RA. For RA, two participants had to be excluded because of technical difficulties with the respiration belt during the recordings or because of the noisy signal recorded. All physiological response channel data were calculated as the change in activity from the baseline level on each trial.

### 2.7 Data analyses

The first step was to conduct the personality profile analyses. Following up on the Bastiaens et al. (2021) procedure, and in order to standardize personality values, we first transformed the data into z‐scores and, then, performed a latent profile analysis (LPA) on Rstudio (version 4.1.0) and R (version 4.1.0) with the mclust package version 5.4.7 (Scrucca et al., 2016). This analysis was performed for both adaptive and maladaptive personality scores separately.

To test for successful emotion induction, we compared the distribution of affective responses during unregulated trials with a 100‐centered (expressivity) or a 0‐centered (all other parameters) distribution. We did this for the full sample and for each of the highlighted profiles. Since we hypothesized a successful emotion induction, we took a one‐tailed *p* value for this test.

For each of the outcome parameters, we then calculated a difference index (DI), defined as the difference between the unregulated condition and the strategies, in the direction directly highlighting the efficiencies of the considered strategies. The DI outcomes were interpreted as not efficient if the results were significantly below 0 because they reveal an increase in emotional arousal. Contrarily, a result above 0 was considered efficient. At last, a nonsignificant difference showed a lack of changes from the unregulated condition. The results were provided by repeated measures ANOVA on IBM SPSS Statistics (version 27), with a within‐subject factor, *Condition* (Reappraisal DI, Suppression DI), and a between‐subjects factor, *Personality* (profiles). The latter analysis was only performed when induction was successful. When the interaction was significant, we performed two post hoc analyses. The first one was an independent *t*‐test for each strategy between the two personality profiles to assess whether the efficiency of a single strategy (reappraisal or suppression) differed between profiles. The second one was a paired *t*‐test, performed to test whether the efficiency of reappraisal and suppression differed within each personality profile. Finally, for each significant result, and in addition to exploring the ANOVA results comparing the different factor levels, we tested whether regulation was efficient by itself, and within each profile when needed. We measured this latter question with one‐sample *t*‐tests.

## 3 RESULTS

### 3.1 Personality profiles

#### 3.1.1 Adaptive personality profiles (NEO‐FFI)

The number of profiles was determined by the interpretation of BIC and AIC values for different models. As Spurk et al. (2020) reported, on a class comparison, the best cluster option is typically the one with the lowest values of BIC and AIC. In this case, we analyzed the possible options from one to five classes (see Table 1, left section). We chose a two‐profile result (BIC value: 1486.46, AIC value: 1444.47).

**TABLE 1:** Class comparison for determination of NEO‐FFI and PID cluster numbers.

	NEO‐FFI		PID	
Class comparison	AIC	BIC	AIC	BIC
1	1462	1489	1462	1489
**2**	**1444**	**1486**	1428	**1470**
3	1448	1506	1430	1488
4	1449	1523	1415	1488
5	1448	1538	**1395**	1484

*Note*: Bold values are the best values per parameter. Nonetheless, the parameter we took mostly into account was BIC (Spurk et al., 2020).

**FIGURE 2:**
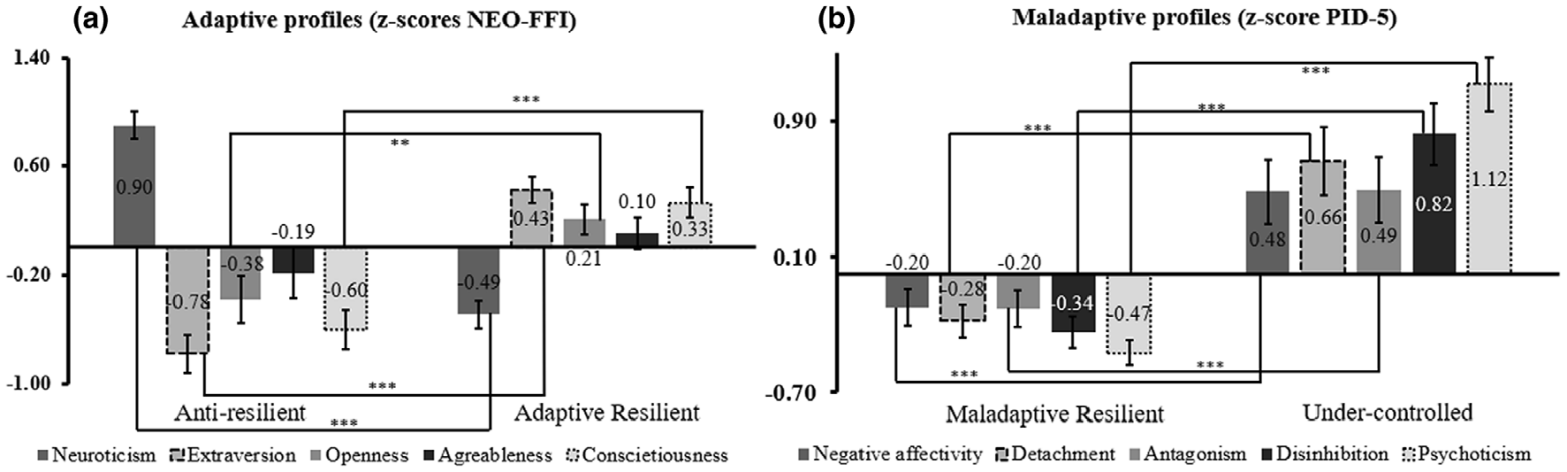
Personality clusters based on the z‐scores of the NEO‐FFI and PID‐5 scores. Panel a: results of adaptive personality profiles based on the NEO‐FFI, with the “Anti‐resilient” and “Adaptive Resilient” profiles. Panel b: results of maladaptive personality profiles based on the PID‐5, with “Maladaptive Resilient” and “Under‐controlled” profiles. Asterisks within or near the bars in both panels represent the independent *t*‐tests of the same trait between profiles, ***p < 0.001, **p < 0.01.

The profiles found can be compared with previous studies (Kerber et al., 2021; Ratchford et al., 2022; Yin et al., 2021). The first profile is labeled as “Anti‐resilient” (*N* = 36), with a higher score of N and lower levels of all other traits; and the second profile is labeled as “Adaptive Resilient” (*N* = 66), with rather lower scores of N and higher levels of all other traits (see Figure 2, Panel a). Between profiles traits were significantly different (*t* = −5.01 to −9.16, *p* = 0.003 to <0.001, *d* = −1.47 to 1.85), except for agreeableness, which was not significant (*t* = −1.40, *p* = 0.166, *d* = −0.29).

#### 3.1.2 Maladaptive personality profiles (PID‐5)

The determination of cluster numbers was done as for the NEO‐FFI. In this case, AIC values directed more toward the 5‐cluster option, while the BIC criterion suggested a two‐class solution (Table 1, right section). We decided to keep the 2‐cluster option (BIC value: 1470.11, AIC value: 1428.11) because of the better homogeneity of the distribution of participants between cluster groups for this solution, as compared to the 5‐class solution.

Referring to Bastiaens et al. (2021), the results led to a “Maladaptive Resilient” cluster (*N* = 72), with low average scores in each maladaptive trait, and an “Under‐controlled” cluster (*N* = 30), with high scores for each maladaptive trait (Figure 2, Panel b). Between profiles, traits were significantly different (*t* = −3.29 to 8.96, *p* = 0.004 to <0.001, *d* = −0.72 to −2.82).

### 3.2 Emotion induction success

To verify that the images successfully induced negative and positive emotional reactions, we performed one‐sample *t*‐tests on the unregulated condition for all parameters. Below (Table 2) is the summary table of the results.

**TABLE 2:** Emotional induction success for each parameter and each valence level.

Emotional response	Neo‐FFI M, Cohen's *d*		PID‐5 M, Cohen's *d*	
	Anti‐resilient	Adaptive resilient	Under‐controlled	Maladaptive resilient
Experience				
Negative	**42.44** *** **, 2.29**	**37.24** *** **, −2.63**	**38.03** *** **, 2.41**	**39.51** *** **, 2.45**
Positive	**35.33** *** **, 2.76**	**35.65** *** **, 2.64**	**36.07** *** **, 2.85**	**35.32** *** **, 2.62**
Expressivity (%)				
Negative	**182.05** *** **, 0.87**	**201.92** *** **, 0.84**	**186.12** *** **, 0.75**	**198.57** *** **, 0.88**
Positive	**179.25** *** **, 0.77**	**162.30** *** **, 1.08**	**150.48** *** **, 1**	**175.70** *** **, 0.90**
Physiological arousal				
HR (Δbpm)				
Negative	0.07, 0.07	**−0.46** ** **, −0.32**	−0.14, −0.13	**−0.32** * **, −0.23**
Positive	−0.13, −0.14	−0.15, −0.19	0.15, 0.17	**−0.26** * **, −0.22**
SCL (ΔμS)				
Negative	**0.04** * **, 0.41**	**−0.05** * **, 0.30**	**0.10** * **, 0.48**	0.02, 0.22
Positive	**−0.03** ** **, −0.50**	−0.02, −0.21	−0.02, −0.21	**−0.02** ** **, −0.36**
RR (Δcpm)				
Negative	**0.02** ** **, 0.55**	0.05, 0.11	**0.16** * **, 0.38**	**0.11** * **, 0.22**
Positive	**0.40** *** **, 0.87**	0.11, 0.21	**0.31** ** **, 0.62**	**0.17** ** **, 0.33**
RA (ΔμV)				
Negative	−0.01, −0.05	**0.03** * **, 0.22**	0.03, 0.24	0.01, 0.04
Positive	−0.05, −0.26	3.03e‐4, 0.001	0.01, 0.04	−0.03, −0.13

*Note*: Bold text represents significant inductions (*p* < 0.05). Test value for one‐sample *t*‐test was 0 for all parameters, except for expressivity where the test value was 100.

Abbreviations: bpm, beats per minutes; cpm, cycles per minute; M, mean.

*** *p* < 0.001;

** *p* < 0.01;

* *p* < 0.05.

### 3.3 General efficiency of reappraisal and suppression

Table 3 shows the emotion regulation efficiency, as measured with the DI, for each response, conditions, and picture valence. At this stage, personality was not taken into account in order to highlight the influence of ER strategies on emotional responses, independently from personality clustering. To analyze this, we ran a repeated measures ANOVA with “*Condition*” as the independent factor.

**TABLE 3:** Efficiency of reappraisal and suppression (DI) on the different parameters.

Emotional response	Mean suppression	Mean reappraisal	Fisher's *F* (*p* value)	*η*_*p*_^2^
Experience				
Negative	−0.002	**8.28**	53.89***	0.34
Positive	−0.61	−5.27	42.41***	−0.29
Expressivity (%)				
Negative	**75.74**	**70.16**	3.39	0.03
Positive	**48.20**	**54.10**	7.48**	0.07
Physiological arousal				
Heart rate (Δbpm)				
Negative	**0.39**	**0.48**	0.45	0.004
Positive	0.16	0.19	0.05	0.001
Skin conductance (ΔμS)				
Negative	0.02	0.02	0.02	0.00
Positive	−0.01	−0.001	1.44	0.02
Respiratory rate (Δcpm)				
Negative	−0.06	0.01	1.5	0.01
Positive	−0.07	−0.03	0.61	0.01
Respiratory amplitude (ΔμV)				
Negative	−0.002	0.02	1.4	0.01
Positive	−0.03	−0.03	0.06	0.001

*Note*: The strategies whose means were significantly above 0, namely efficient, are represented in bold text. Note that a decrease in positive experience following regulation was labeled as not efficient, even if this was significant.

*** *p* < 0.001;

** *p* < 0.01.

After this overview of emotion induction and the general efficiency of ER strategies regardless of personality, we decided to concentrate the analyses only on the parameters that resulted significant in the emotional induction (see Table 2). Thus, from this moment on, analyses will concentrate only on a few parameters. For experience and expressivity, we considered all the parameters, since all our trials were successful in inducing changes in these channels. For physiological parameters, however, we further conducted analyses for HR during negative viewing in Adaptive Resilient profile and in both valences for Maladaptive Resilient group; for SCL during negative viewing in both NEO‐FFI profiles and in Under‐controlled PID‐5 profile, while during positive viewing in Anti‐resilient (NEO‐FFI) and Maladaptive Resilient (PID‐5) profiles; for RR during both negative and positive viewings in Anti‐Resilient profile (NEO‐FFI) and both valences in all maladaptive profiles (PID‐5); and for RA during negative viewing in Adaptive Resilient profile.

### 3.4 Strategy efficiency depending on personality profiles

To analyze how adaptive and maladaptive personalities influence ER efficiency, we ran repeated‐measure ANOVAs, with “Condition” as the within‐subject factor and “Personality” as the between‐subject factor.

Table 4 shows the efficiency of ER strategies depending on personality profiles. In this table, we represented the strategy efficiency within the personality profile. In contrast to Table 3, which focused on the general efficiency of strategies, regardless of personality profiles, Table 4 shows the efficiency of each strategy in each personality profile. In this way, it was possible to highlight which strategy was efficient and which was not in the different personality profiles, before turning to investigate whether this efficiency was different between profiles (see Sections 3.4.1 and 3.4.2). The analysis was made with one‐sample *t*‐test and data were DI values.

**TABLE 4:** Efficiency of strategies depending on personality profiles.

Emotional response	NEO‐FFI M, Cohen's *d*				PID‐5 M, Cohen's *d*			
	Anti‐resilient		Adaptive resilient		Under‐controlled		Maladaptive resilient	
	Reappraisal	Suppression	Reappraisal	Suppression	Reappraisal	Suppression	Reappraisal	Suppression
Experience								
Negative	**6.17** *** **, 0.61**	−0.30, 0.04	**9.43** *** **, 0.72**	0.16, 0.02	1.80, 0.22	−2.36*, −0.47	**10.98** *** **, 0.89**	0.98, 0.11
Positive	−3.45*, −0.38	1.28, 0.19	−6.25***, −0.65	0.24, 0.03	−1.04, 0.15	1.90, 0.34	−7.03***, −0.71	0.08, 0.01
Expressivity (%)								
Negative	**63.73** *** **, 0.66**	**73.60** *** **, 0.71**	**74.35** *** **, 0.74**	**76.63** *** **, 0.77**	**57.07** ** **, 0.59**	**64.38** ** **, 0.64**	**76.24** *** **, 0.76**	**80.22** *** **, 0.79**
Positive	**53** ** **, 0.59**	**62.29** *** **, 0.60**	**45.60** *** **, 0.88**	**49.64** *** **, 0.93**	**36.52** *** **, 0.78**	**45.80** *** **, 0.93**	**53.07** *** **, 0.72**	**57.57** *** **, 0.70**
Physiological arousal								
Heart rate (Δbpm)								
Negative			**0.36** * **, 0.25**	0.33, 0.23			**0.63** *** **, 0.40**	**0.52** ** **, 0.32**
Positive							0.18, 0.11	0.14, 0.09
Skin conductance (ΔμS)								
Negative	0.01, 0.08	0.03, 0.35	0.02, 0.16	0.01, 0.08	0.02, 0.20	0.03, 0.29		
Positive	0.01, 0.06	−0.01, −0.15					0.01, 0.09	−0.01, −0.1
Respiratory rate (Δcpm)								
Negative	0.04, 0.11	0.05, 0.1			0.04, 0.1	−0.08, −0.14	−0.003, −0.01	0.04, 0.1
Positive	0.07, 0.13	0.09, 0.20			0.18, 0.32	0, 0	−0.11, −0.21	−0.10, −0.17
Respiratory amplitude (ΔμV)								
Negative			0.02, 0.12	0.03, 0.18				
Positive								

*Note*: Efficiency of each strategy in both adaptive and maladaptive personality profiles. Efficiency indications are evidenced in bold.

Abbreviation: M, mean.

*** *p* < 0.001;

** *p* < 0.01;

* *p* < 0.05.

#### 3.4.1 Five‐Factor Model

##### Experience

**FIGURE 3:**
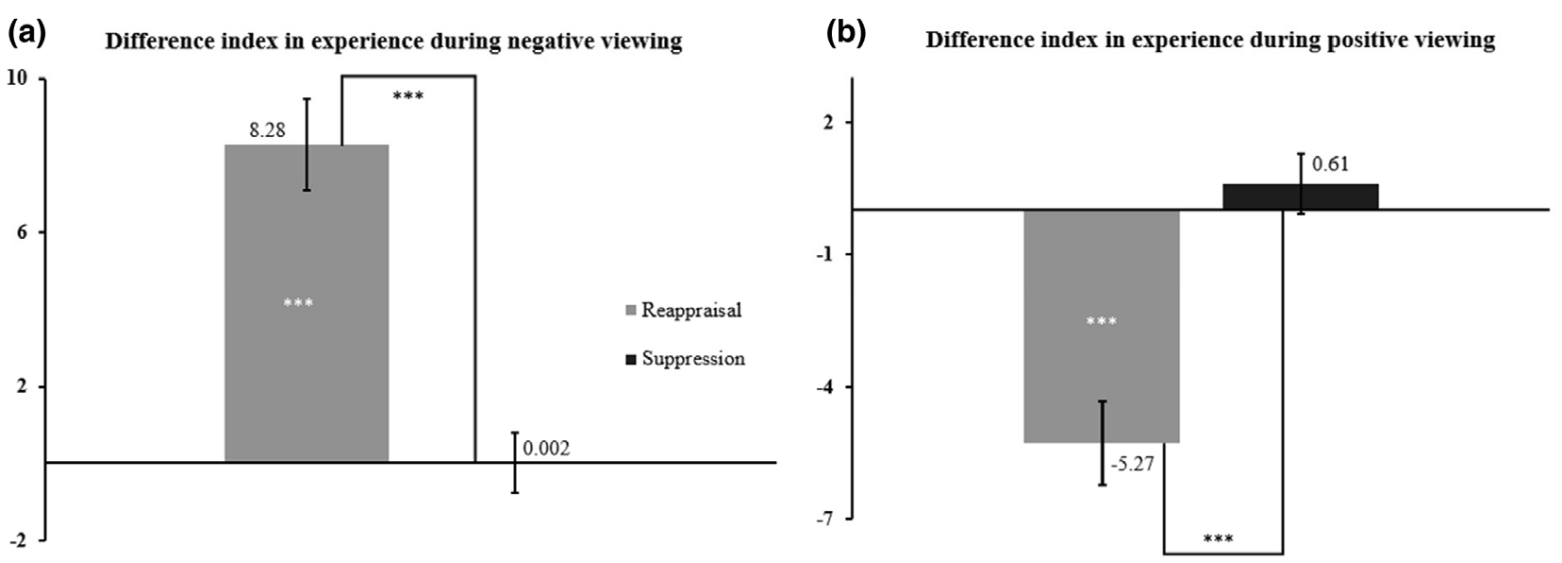
DI of experience during negative viewing (Panel a) and during positive viewing (Panel b). Dispersion is indicated with Standard Errors of the Mean. The lines between the conditions in the two panels indicate the difference between reappraisal and suppression. The asterisks within the bars in both panels represent the one‐sample *t*‐test results, ***p < 0.001.

During negative viewing, the DI for experience showed a significant result of Condition, *F*_(1;100)_ = 44.72, *p* < 0.001, *η*_*p*_^2^ = 0.30 (Figure 3, Panel a), but the main effect of Personality, *F*_(1;100)_ = 1.13, *p* = 0.289, and the interaction between Personality and Condition, *F*_(1;100)_ = 1.40, *p* = 0.239, were not significant.

During positive viewing, the main effect of Condition was significant, *F*_(1;100)_ = 35.36, *p* < 0.001, *η*_*p*_^2^ = 0.26 (Figure 3, Panel b), but the effect of Personality, *F*_(1;100)_ = 1.80, *p* = 0.182, and the interaction, *F*_(1;100)_ = 0.87, *p* = 0.354, were not significant.

##### Expressivity

**FIGURE 4:**
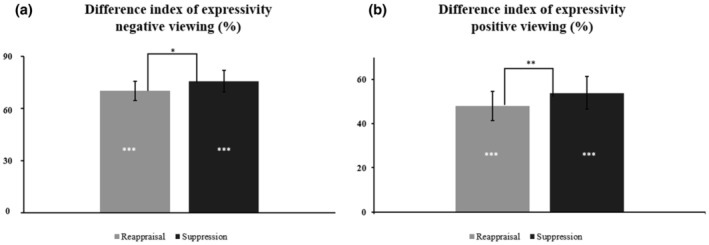
DI of expressivity, measuring strategy efficiency, during negative viewing (Panel a) and positive viewing (Panel b). Dispersions are indicated with Standard Errors of the Mean. The lines between the conditions indicate the difference between reappraisal and suppression. The asterisks within the bars in both panels represent the post hoc analyses measured with one‐sample *t*‐tests, ***p < 0.001, **p < 0.01, *p < 0.05.

During negative viewing, the main effect of Condition, *F*_(1;100)_ = 4.68, *p* = 0.033, *η*_*p*_^2^ = 0.04 was significant (Figure 4, Panel a). However, the main effect of Personality, *F*_(1;100)_ = 0.11, *p* = 0.741, and the Condition × Personality interaction, *F*_(1;100)_ = 1.82, *p* = 0.180, yielded no significant results.

During positive viewing, the main effect of Condition was found to be significant, *F*_(1;100)_ = 8.77, *p* = 0.004, *η*_*p*_^2^ = 0.08 (Figure 4, Panel b). The main effect of Personality, *F*_(1;100)_ = 0.47, *p* = 0.492, and the interaction effect, *F*_(1;100)_ = 1.35, *p* = 0.247, were, however, nonsignificant.

##### Physiological arousal

The results for SCL during negative viewing were not significant: for the Condition effect, *F*_(1;81)_ = 0.16, *p =* 0.689, the Personality effect, *F*_(1;81)_ = 0.19, *p =* 0.659, and the interaction effect, *F*_(1;81)_ = 1.04, *p* = 0.311, as well as for the difference between conditions for the positive viewing in SCL in the Anti‐resilient profile, *t*_(29)_ = 1.45, *p* = 0.157.

To assess ER efficiency, we required an emotional reaction, in order to be able to see its modulation with regulation. For the remaining physiological arousal parameters (HR, RR, and RA), we hence focused the exploration of ER efficiency only on profiles and valences that showed a significant emotional induction (see Table 2). This was done with paired *t*‐tests. For HR during negative viewing in the Adaptive Resilient profile, contrasts between conditions were also nonsignificant, *t*_(65)_ = 0.18, *p* = 0.858. For RR in both valences for the Anti‐resilient profile, difference between conditions were still not significant; during negative, *t*_(33)_ = −0.174, *p* = 0.863, and positive viewing, *t*_(33)_ = −0.25, *p* = 0.806. Finally, the difference between strategies for RA during negative viewing for the Adaptive Resilient profile also presented no significant result, *t*_(64)_ = −0.494, *p* = 0.623.

#### 3.4.2 Maladaptive Personality Trait Model

##### Experience

For experience during negative viewing, Condition, *F*_(1;100)_ = 34.32, *p* < 0.001, *η*_*p*_^2^ = 0.26, the main effect of Personality, *F*_(1;100)_ = 13.01, *p* < 0.001, *η*_*p*_^2^ = 0.11 and the Condition × Personality interaction, *F*_(1;100)_ = 5.84, *p* = 0.017, *η*_*p*_^2^ = 0.05 were significant.

**FIGURE 5:**
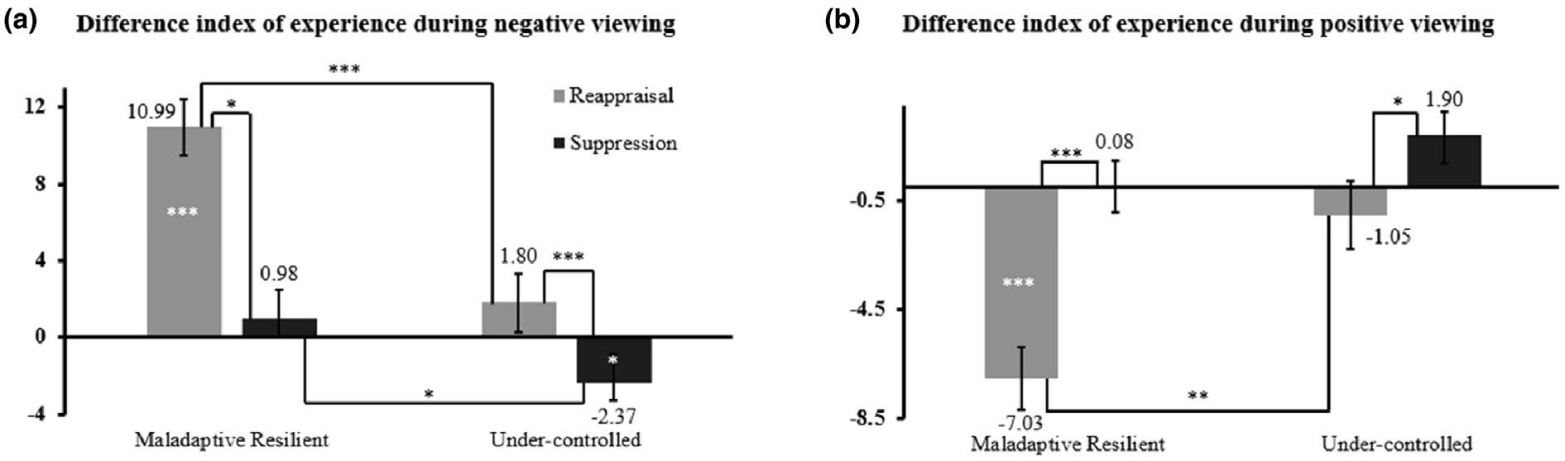
DI on experience during negative (Panel a) and positive (Panel b) viewing for each MPTM profile. Lines between conditions and between groups in both panels indicate the significant difference between reappraisal and suppression or a single strategy difference between groups. Dispersions are indicated with Standard Errors of the Mean. The asterisks within or near the bars in both panels represent the one‐sample *t*‐test results, ***p < 0.001, **p < 0.01, *p < 0.05.

The post hoc tests for the interaction indicated a significant difference between the two profiles, for reappraisal, *t*_(100)_ = −3.73, *p* < 0.001, *d* = −0.81, as well as for suppression, *t*_(100)_ = −1.96, *p* = 0.018, *d* = −0.43 (Figure 5, panel a). Reappraisal and suppression yielded different efficiency levels in the Maladaptive Resilient profile, *t*_(71)_ = −7.05, *p* < 0.001, *d* = −0.83, and in the Under‐controlled profile, *t*_(29)_ = −2.68, *p* = 0.012, *d* = −0.49. In the Maladaptive Resilient profile, reappraisal was efficient, *t*_(71)_ = −7.54, *p* < 0.001, *d* = −0.89, but suppression was not, *t*_(71)_ = −0.95, *p* = 0.344. In the Under‐controlled group, reappraisal was not efficient, *t*_(29)_ = −1.20, *p* = 0.241, while suppression yielded significant results, *t*_(29)_ = 2.57, *p* = 0.016, *d* = −0.22, but indicating a reinforcement of experience during the viewing of negative pictures.

Similar results appeared in positive viewing, with significant main effects of Condition, *F*_(1;100)_ = 26.62, *p* < 0.001, *η*_*p*_^2^ = 0.21, Personality, *F*_(1;100)_ = 7.16, *p* = 0.009, *η*_*p*_^2^ = 0.07 and the Condition × Personality interaction, *F*_(1;100)_ = 4.55, *p* = 0.035, *η*_*p*_^2^ = 0.04 (Figure 5, panel b).

The post hoc tests following the interaction showed for reappraisal a significant difference between groups, *t*_(100)_ = 3.03, *p* = 0.001, *d* = 0.66, whereas for suppression the difference between groups was not significant, *t*_(100)_ = 1.21, *p* = 0.227. The subsequent paired and one‐sample *t*‐tests showed that, for the Maladaptive Resilient profile, there was a significant difference between the strategies, *t*_(71)_ = 6.12, *p* < 0.001, *d* = 0.72, reappraisal significantly decreasing positive emotions, *t*_(71)_ = 6.06, *p* < 0.001, *d* = 0.71, while suppression did not, *t*_(71)_ = −0.09, *p* = 0.929. For the Under‐controlled profile, we also noted a difference between strategies, *t*_(29)_ = 2.50, *p* = 0.016, *d* = 0.47, but, this time, neither reappraisal, *t*_(29)_ = 0.83, *p* = 0.414, nor suppression, *t*_(29)_ = −1.85, *p* = 0.074 triggered significant changes in the positive experience.

##### Expressivity

During negative viewing, Condition, *F*_(1;100)_ = 3.62, *p* = 0.060, the main effect of Personality, *F*_(1;100)_ = 0.66, *p* = 0.418, and the interaction, *F*_(1;100)_ = 0.31, *p* = 0.575, showed nonsignificant results.

During positive viewing, only the main effect of condition was found significant, *F*_(1;100)_ = 8.47, *p* = 0.004, *η*_*p*_^2^ = 0.08 (see Figure 4, panel b), whereas the main effect of Personality, *F*_(1;100)_ = 0.86, *p* = 0.355, and the Condition × Personality interaction, *F*_(1;100)_ = 1.02, *p* = 0.315, were not significant.

##### Physiological arousal

As in the case of the FFM analyses, we conducted paired *t*‐tests contrasting ER strategies efficiency in HR and SCL for profiles and valences that showed a successful induction. In HR during negative viewing, strategies used by the Maladaptive Resilient profile yielded no difference, *t*_(71)_ = 0.71, *p* = 0.482, similarly as for HR during positive viewing, *t*_(72)_ = 0.20, *p* = 0.838. In SCL, results for the Maladaptive Resilient profile in positive viewing and for the Under‐controlled profile in negative viewing did not show difference between strategies *t*_(56)_ = 1.37, *p* = 0.175, and *t*_(25)_ = −0.29, *p* = 0.774, respectively. Analysis for RR during negative viewing showed no main effects of Condition, *F*_(1;94)_ = 1.75, *p* = 0.190, Personality, *F*_(1;94)_ = 0.02, *p* = 0.900, as well as no significant interaction *F*_(1;94)_ = 0.29, *p* = 0.590. Similar results were found in positive viewing, where the main effects of Condition, *F*_(1;94)_ = 1.88, *p* = 0.174, and Personality, *F*_(1;94)_ = 2.89, *p* = 0.092, were not significant. The interaction *F*_(1;94)_ = 2.47, *p* = 0.119, was also not significant.

## 4 DISCUSSION

The main purpose of this study was to evaluate the efficiency of reappraisal and suppression strategies in association with adaptive and maladaptive personality profiles by investigating them on every emotional response (experience, expressivity, and physiological arousal). Previous studies have recognized that the ER's efficiency may depend on factors such as context or personality (Gross et al., 2006; Purnamaningsih, 2017), but there was still a lack of literature in the study of personality profiles associated with ER's efficiency, and particularly that of reappraisal and suppression, despite the extensive work devoted to the understanding of the differences between these two strategies.

In our search of different profiles, and regarding the FFM profiles (see Section 3.1.1), we found two main profiles: the Adaptive Resilient and Anti‐resilient profiles. This result is consistent with Yin et al. (2021) and Ratchford et al. (2022), where these two profiles were described in a similar way. Concerning the MPTM model (see Section 3.1.2), two personality profiles were found: the Maladaptive Resilient and the Under‐controlled profiles. These profiles confirmed the results of Bastiaens et al. (2021) and Rossi et al. (2021). As Rossi et al. (2021) suggested, accurately labeling and describing maladaptive profiles may be particularly important because it may lead to treatment differentiation between patients. Depending on their membership in a certain profile, patients may benefit from a more tailored treatment that could potentially increase the success of a therapy.

When considering ER efficiency, and focusing on adaptive personality, differences of strategies per profile were not confirmed for either valence but, overall, we found differences between strategies in the regulation of experience. The same is true for expressivity, where differences between the profiles were not found. For physiological arousal, our hypotheses were rejected and no significant difference between strategies or between profiles was found. Several points could be highlighted regarding these results (see Section 4.1). Regarding maladaptive personality profiles, we found on the one hand some differences in efficiency between profiles regarding experience, confirming our hypothesis. On the other hand, the hypotheses concerning expressivity and physiological arousal were not confirmed and no differences between the profiles were found. Several details of the findings, however, deserve to be discussed in more details (see Section 4.2).

### 4.1 Adaptive personality profiles and ER efficiency

When looking at the adaptive personality profiles, and with respect to experience during negative viewing (Figure 3, Panel a), only reappraisal reduced negative reactions, and this in both profiles (see Table 4). On the contrary, suppression had no influence in decreasing experience in front of negative pictures. The result regarding reappraisal is consistent with previous studies (Gross et al., 2006; Zaehringer et al., 2020) in which reappraisal was indicated as beneficial in reducing negative subjective feelings. However, it is interesting to note that, whereas Adaptive Resilient people benefited from the positive impact of reappraisal, as expected, Anti‐Resilient people also retrieved benefits from using this strategy. This goes in contrast with Purnamaningsih (2017) findings, which showed that people with high levels of N are less likely to benefit from reappraisal effects. About suppression, this result confirms that it is not suitable for reducing negative feelings (Gross & Levenson, 1997). In this case, personality did not influence the efficiency of suppression, presenting the same conclusions we had when personality was not taken into account (Trentini & Dan‐Glauser, 2023).

Regarding experience in positive viewing during reappraisal, experience values were lower in both profiles. Suppression behaved similarly to what observed for negative viewing, without triggering either an increase or a decrease in the positive experience. During reappraisal, experience levels probably decreased regardless of emotional valence type (Kim & Hamann, 2007; Meyer et al., 2012). Furthermore, these results indicate that this phenomenon may not be influenced by personality. This is particularly interesting for Adaptive Resilient profile. Unlike the predictions, the inability of reappraisal to discriminate between negative and positive valence led Adaptive Resilient people to not benefit from reappraisal's effects when considering positive situations. Suppression results are consistent with Kalokerinos et al. (2015) and Meyer et al. (2012), who reported that suppression did not significantly change experience during positive viewing in comparison to the unregulated condition.

Overall, on the one hand, our hypothesis that reappraisal would have been more efficient than suppression in regulating negative emotions has been confirmed. On the other hand, during positive viewing, reappraisal was not able to increase positive emotions, and neither was suppression, rejecting our hypothesis. In this channel, the influence of personality on ER efficiency was not confirmed.

Regarding expressivity, during both negative and positive viewing, we found a general efficiency of both strategies. In expressivity during negative viewing, suppression resulted in a better performance than reappraisal in decreasing negative reactions. However, in association with personality, we did not detect significant differences in the two strategies between personality profiles. We can therefore assume that both strategies may influence expressivity, both on negative and positive emotions, and that belonging to a certain personality group does not preclude the influence of strategies on expressivity. This partially confirmed our expectations. On one side, we showed a better efficiency of suppression in the expressivity, but on the other side, the belongingness to a personality profile has not influenced the result.

Physiological parameters did not show many significant results in adaptive profiles. Only during negative viewing on HR, reappraisal proved to be efficient in reducing the emotional impact of the images in Adaptive Resilient profile (see Table 4), leading to similar results of Zaehringer et al. (2020) and Mohammed et al. (2021). Some other parameters, such as SCL and RA, presented no significant results. For SCL, the lack of results can be coherent with Mohammed et al. (2021), but, overall, this situation leads us to hypothesize that there could be a differential influence of reappraisal on physiological parameters. Indeed, it may be possible that reappraisal may have a greater impact on some parameters as compared to others, creating a disparity in the physiological responses. Altogether, our hypothesis about physiological arousal was confirmed, since reappraisal was efficient in HR in people low in N. Nonetheless, this could be considered as a partial confirmation due to the lack of efficiency in the other parameters.

### 4.2 Maladaptive personality profiles and ER efficiency

When looking at the maladaptive personality profiles, and regarding experience during negative viewing, reappraisal significantly decreased negative feelings in the Maladaptive Resilient group but not in the Under‐controlled group, whereas suppression had no impact on either personality group (Figure 5, Panel a). These results highlight the efficiency of reappraisal in the Maladaptive Resilient group and the inability to do the same in the Under‐controlled group. This may happen because Maladaptive Resilient individuals may have more cognitive resources than Under‐controlled individuals. This is consistent with Rogier et al. (2020), who suggested that impulsive people (a feature of Under‐controlled profile, Sârbescu & Boncu, 2018), have more difficulties in implementing reappraisal, but not with Opoka, Ludwig, et al. (2021), who presented that reappraisal was effective in clinical people with high *psychoticism* trait levels. However, the clinical sample of the latter study followed a medical treatment that could have indirectly improved their overall access to ER. Furthermore, they were motivated to participate in an emotion‐focused trial, which could have led to an involuntary selection of patients with better ER abilities. As said before, suppression during negative viewing resulted nonsignificant for Maladaptive Resilient people. Considered that Maladaptive Resilient group is one of the most “adaptive” profiles in the maladaptive personality (Rossi et al., 2021), it implies that they may be more rich in cognitive resources. The easier access to these resources may lead to a propensity toward the application of more adaptive strategies (like reappraisal), and this can be reflected in the ER efficiency. For the Under‐controlled individuals, we can hypothesize that suppression may not be powerful enough to efficiently regulate emotions, and this is also consistent with the results of Rogier et al. (2020). As Borges and Naugle (2017) reported in their study on PD clusters, people from cluster B (characterized by impulsivity and *Disinhibition*) were found to be negatively related to suppression. It may be that, in our case, and despite the instruction to suppress emotions, Under‐controlled people may not be able to implement this strategy. For this group, neither reappraisal nor suppression were good strategies for regulating negative emotions, and we therefore cannot suggest the application of any of these strategies for this group. It is possible then that some other ER strategies may be more adequate for this profile. Considered their difficulties in reinterpreting the situation (Webb et al., 2018) and maintaining self‐control (Rossi et al., 2021), situation selection may be a possible alternative for Under‐controlled people to successfully regulate emotions. Indeed, this strategy seems to best fit people with poor resources for regulating emotions and being impulsive at the same time (Webb et al., 2018). In this way, they could strive for the fastest path to a pleasant emotion without the need for high competences in ER. Another possible strategy may be distraction. Given the impulsive nature of Under‐controlled people (Sârbescu & Boncu, 2018), this strategy may help them to easily get away from negative emotions and focus on positive ones. However, this still needs to be analyzed. Returning to our hypotheses on experience for maladaptive personalities, the results led to a partial confirmation of them. Indeed, reappraisal during positive viewing was not efficient in people high in *Negative Affectivity*. Despite this, our expectations about experience during negative viewing were rejected since the efficiency of reappraisal during negative viewing resulted better in Maladaptive Resilient people.

Concerning negative expressivity, none of the parameters showed significant differences between maladaptive profiles for each strategy, rejecting our hypotheses about these parameters. Moreover, as shown in Table 4, both strategies were significant within both profiles, meaning that both strategies are able to influence expressivity, but in an equal fashion. Positive expressivity results were similar to what we had for adaptive personality and, overall, we can presume that only ER strategies may actually play a role in this emotional response.

Regarding physiological arousal, our hypotheses were confirmed only for HR, since we expected a reduction in physiological arousal in people low in maladaptive traits during reappraisal. Indeed, as Table 4 presented, reappraisal and suppression resulted efficient in the Maladaptive Resilient profile in modulating HR when confronted to a negative situation. This confirms the main advantage of Maladaptive Resilient profile, who can adaptively face stressful stimuli (Bastiaens et al., 2022), probably due to the overall low values of maladaptive traits and the better management of emotion regulation, in comparison with other maladaptive profiles (Bastiaens et al., 2021). Of note, the efficiency of suppression in decreasing HR goes in contrast to Gross (1998), where an increase in HR during this strategy was found. This discrepancy between results may come from the differential timing of trials, that is, around 1‐min trial for Gross (1998) versus 8 s in our study. As we previously reported (Trentini & Dan‐Glauser, 2023), over a short interval, suppression can turn out to be efficient, and therefore a possible rebound caused by this strategy in HR may go undetected.

Despite these new insights about HR during negative viewing, it is important to note the absence of significant results for the other physiological parameters, in each personality profile and ER strategy, which rejoin the results found in the analyses of the adaptive profiles. This scarce effect of regulation on physiological parameters calls for a deeper analysis of the emotional arousal in the different profiles and the analyses of what is to be regulated for each, and, consequently, of the choice of the ER strategies for regulating the physiological arousal for our particular participants. This will be further discussed in the next paragraph.

### 4.3 Specificities of physiological arousal and regulation in adaptive and maladaptive profiles

Despite the few significant results retrieved from physiological parameters on both personality profiles (see Sections 4.1 and 4.2), there are some other important points regarding physiological arousal that deserve to be discussed, both regarding the reactivity aspect and the regulatory one.

As seen in Table 2, not all emotion responses were sensitive to induction for all parameters and all groups but, interestingly, for the same induction stimuli, there were some reactivity specificities depending on personality. If emotion induction was reflected in emotional experience and expressivity for all profiles and all valences, we noticed differential emotional response patterns depending on the personality for the physiological parameters. Concerning the adaptive profiles in the Anti‐resilient group, emotional responses can be noticed in SCL and RR, while in the Adaptive Resilient group, induction triggered changes in a negative context only, visible in HR, SCL, and RA parameters. Similar things happened in the maladaptive profiles. In the Under‐controlled profile, the emotional induction was reflected in the changes of RR for both negative and positive trials, and in SCL for negative trials. On the contrary, in the Maladaptive Resilient profile, response changes following induction were reflected in HR, and RR for both trial valences and in SCL for positive trials. The absence of HR results in the Under‐controlled profile, and the presence of these in the Maladaptive Resilient profile, highlight that probably Maladaptive Resilient people may be more reactive in HR than Under‐controlled ones. At the same time, the lack of RA results in both profiles suggests that this parameter may not be adequate to detect physiological differences between these personality profiles. For these reasons, we may hypothesize that, depending on the studied parameter, personality may show more or less influence on the physiological result. This hypothesis may be supported by Herpertz et al. (2001). In their study, they compared emotions in criminals with BPD and antisocial behaviors and noncriminal people by showing them positive, neutral or negative images. They found significant lower SCL values in the criminal group in all emotional valences. This result suggests that personality may lead to differential reactivity (in Herpertz et al., 2001, a case of hypo reactivity) of some physiological parameters. Based on these examples, we could thus make the hypothesis that there exists a differentiated pattern of induction between profiles. The fact that this happens in physiological parameters leads to think that these parameters may be more sensitive to this particular experimental setup (short image viewing) and to the underlying differences between personality groups. This may be consistent with Wainio‐Theberge and Armony (2023), who found individual differences in the bodily sensations toward emotions. Thus, it may be possible that some personality profiles or tendencies could influence the physiological impact of emotions and this particular statement definitely deserves to be further explored.

The second piece of information retrieved by our results is that even ER strategies seem to have a differential influence on physiological parameters. This is visible in both adaptive and maladaptive personality models. In Adaptive Resilient profile, reappraisal was efficient in diminishing the impact of negative images on HR but not on other parameters like SCL and RA. In Maladaptive Resilient profile, both strategies had an impact again on HR but not on other physiological values. However, Anti‐resilient and Under‐controlled profiles presented no efficiency, neither in reappraisal nor in suppression. Actually, in HR, both profiles presented the absence of emotional induction, highlighting the clear contrast with the other profiles. This is also similar to Herman et al. (2018), which presented a blunted cardiac reaction to stress in people high in impulsivity (a common characteristic of Anti‐resilient and Under‐controlled people). So, the personality variable seen in the emotional induction plays again a role, creating an additional interaction with ER on the final outcome.

In summary, these results concerning adaptive and maladaptive personalities suggest a complex interaction between personality, the physiological parameters involved, and the ER strategy at play, which ultimately determines the final emotional response. In our case, for most parameters, emotions were not induced in all clusters, implying that personality has an influence on the degree of induction and that, consequently, some parameters were not activated enough to detect a significant difference from baseline. This could be resulting from the method used in the present study or driven by the intrinsic hypo‐activation of certain channels for certain personalities. Furthermore, in some parameters (such as SCL during negative viewing in adaptive personality and RR during negative viewing in maladaptive personality), where emotions were successfully induced, irrespective of the personality belonging, the influence of the two ER strategies that we chose for this study was not enough to highlight a difference between them. This case leads us to think that both personality and strategies can modify and have a significant impact on the intensity of the physiological response. Future research on the efficiency of other strategies may help to disentangle this phenomenon and give more information about how and in which parameters, and which strategies amplify (or blunt) the physiological reaction. Thus, in order to detect, or provoke, an efficient response, one needs a perfect match between personality, ER, and parameters. Therefore, to clearly detect the final regulated emotional physiological response, it is important to consider a few elements. First, it is important to characterize which physiological parameters are activated by which personalities. Next, it needs to be analyzed whether there are differences between strategies within a given profile (as we were able to show in HR for the Adaptive Resilient profile) and within a single strategy between profiles. Third, it should be taken into account which parameter can be more adequate to detect the efficiency of ER strategies because, from our results, we can see that they may have an impact on some parameters but not on others. Hence, the differential reactivity and potential differential efficiency of given ER strategies on some parameters for the different profiles depict a terribly complex configuration that will definitely require many additional studies. As a first step, it would be interesting to further investigate the impact of personality and ER strategies on physiological parameters in order to understand which parameter is more adequate to measure efficiency. In addition, further pushing the assessment of this interplay would play a role in a clinical context. These studies may indeed help clinicians to understand which ER strategies to work on, the ones that could be more effective depending on symptoms, or which additional therapeutic elements can favor psychological improvement, depending on the match between ER strategies, personalities, and physiological channels.

### 4.4 Limitations and future directions

Considering that this study is one of the first attempt to link the efficiency of ER strategies to personality profiles, some limitations need to be considered in the future.

Our sample consisted of undergraduate psychology students. This could have brought some educational biases and made the results less generalizable to other groups of the population. Furthermore, studying psychology may have prompted participants to be more aware of emotion regulation processes than the general population would be. Therefore, it would be interesting in the future to include participants outside psychology faculty and outside the university in order to expand the generalizability of the findings and diminish the influencing aspects that characterize people in the academic field. Regarding the sample, we could also highlight that, despite being within the range of acceptable sample size for LPA (Campez et al., 2020) the determination of profiles was based on a sample size close to the lower threshold. Future studies would benefit from having a larger sample size to increase the likelihood of obtaining more fine‐grained personality profiles. Our three‐step procedure (LPA, classification, and testing of emotional variables) may alert with respect to classification errors that could bias our conclusion with respect to the emotional processes into play. As such a method was more appropriate to our sample and exploratory approach, we renounced to correct for such classification bias, but future studies could consider testing LPA outcomes of personality and ER efficiency with alternative methods like the Bolck–Croon–Hagenaars approach (Bakk & Kuha, 2021). As Bauer (2022) reported, these methods could control the classification uncertainty given by LPA models and increase the accuracy of the results provided by the secondary analyses.

In order to let participants implement the required strategy, we used validated instructions for regulating emotions and checked with participants their correct understanding of the strategies. Despite this, we cannot be completely sure that participants correctly executed the given instructions. In addition, it is likely that participants implicitly and unconsciously used other ER strategies that we were not aware of (Gyurak et al., 2011), both in the unregulated and regulated conditions. Future studies could be more cautious with this aspect by, for example, quantifying to what extent they were able to perform the task. Finally, as shown particularly by the results of the maladaptive profiles, reappraisal and suppression seemed to efficiently influence some emotion responses but not all of them. Therefore, studies of other ER strategies are needed to assess which strategy may be more beneficial for which type of personality profile and on which channel. Finally, it could be interesting to analyze some hypotheses we made throughout the discussion section, especially the one targeting the physiological reactivities of the different profiles and the differential effect on these parameters that we found for the different ER strategies.

## 5 CONCLUSION

In this study, we aimed to compare the efficiency of reappraisal and suppression with personality profiles, to identify whether certain strategies are more suitable for certain personalities. To do this, we used an innovative methodological approach, the difference index, which allowed us to better target the impact of the strategies on the studied parameters. The determination of personality profiles confirmed the results of previous studies (Bastiaens et al., 2021; Yin et al., 2021). Particularly in the maladaptive personality, reappraisal and suppression showed different results depending on the profile. Reappraisal was responsible for the decrease in negative emotions during experience and of changes in HR in Maladaptive Resilient profile, but not for the Under‐controlled profile regarding experience. This suggests that this strategy may be particularly appropriate for Maladaptive Resilient people to decrease negative emotions. Always on maladaptive profiles, suppression was not effective or did not influence enough experience values in either profile or valence. This suggests that this strategy is not ideal in either profile for decreasing negative emotions. In adaptive and maladaptive personalities, both strategies were efficient in decreasing negative expressivity and did not show differences between profiles. Emotion induction did not result in a noticeable physiological response in many parameters. More interestingly, we show that different profiles reacted differently to the same emotion material, with differential physiological changes. With such differences in reactivity, differences between strategies and between profiles were hard to highlight. This study nevertheless provided new information in the field of personality and ER research. We believe that these initial findings may prompt future studies to continue this examination in order to fill this gap and give more insights for clinical and nonclinical psychological treatments.

## AUTHOR CONTRIBUTIONS

E.T: Software, formal analysis, investigation, data collection, data curation, writing and visualization. E.D‐G: Conceptualization, methodology, software, writing, visualization, supervision, project administration, and funding acquisition.

## FUNDING INFORMATION

This work was supported by a Swiss National Science Foundation Eccellenza Grant (no PCEFP1_186836) to E.D‐G.

## CONFLICT OF INTEREST STATEMENT

The authors declare that they have no known competing financial interests or personal relationships that could have appeared to influence the work reported in this paper.

## ETHICS STATEMENT

This study protocol has been approved by the UNIL‐SSP ethics committee (approval number: C_SSP_112020_00006).

## Data Availability

Data for this manuscript are available at https://osf.io/pe9n6/?view_only=60708aa1e4cb4a6693096e338545b8d2.
